# Senear–Usher Syndrome or Coexistence of SLE with Pemphigus Vulgaris—A Case Report with Literature Review

**DOI:** 10.3390/jcm14020409

**Published:** 2025-01-10

**Authors:** Magdalena Kutwin, Marcelina Kądziela, Tomasz Stein, Marzena Kraska-Gacka, Anna Woźniacka, Agnieszka Żebrowska

**Affiliations:** 1 Department of Dermatology and Venereology, Medical University of Lodz, pl. Hallera 1, 90-647 Lodz, Poland; magdalena.kutwin@umed.lodz.pl (M.K.); marcelina.kadziela@stud.umed.lodz.pl (M.K.); marzena.kraska@umed.lodz.pl (M.K.-G.); anna.wozniacka@umed.lodz.pl (A.W.); 2 Department of Dermatology, Poznan University of Medical Sciences, 60-806 Poznan, Poland; t.stein@wp.pl

**Keywords:** pemphigus erythematosus, Senear–Usher syndrome, pemphigus vulgaris, SLE

## Abstract

Senear–Usher syndrome, or pemphigus erythematosus (PE), is a rare autoimmune disorder characterized by the coexistence of features from both lupus erythematosus (LE) and pemphigus foliaceus (PF). We describe a 41-year-old patient initially diagnosed with cutaneous and then systemic lupus erythematosus (SLE), who after a few years developed new skin lesions: erythematous and erosive eruptions partially covered by crusts located on the trunk and flaccid blisters on the extremities. Direct immunofluorescence of perilesional skin revealed deposits of IgG in the intercellular space of the epidermis and granular deposits of C3 at the dermo–epidermal junction. Additional testing, revealing autoantibodies against the intercellular space of the epidermis, and direct immunofluorescence (DIF) examination allowed a diagnosis of pemphigus vulgaris coexisting with lupus. Further, DIF study revealed granular deposits of immunoglobulin G (IgG) in the intercellular spaces of the epidermis and granular deposits of the C3 along the basement membrane. Clinical appearance led to suspicion of Senear–Usher syndrome. in this patient. This case report explores the diagnostic challenges posed by the patient’s overlapping symptoms and immunological findings, suggesting an infrequent manifestation of Senear–Usher syndrome or a combination of SLE and pemphigus vulgaris. The case highlights the complexity of chronic inflammatory skin diseases and the need for tailored treatment approaches in such cases. Despite temporary improvement, the patient experienced relapses. We performed a descriptive literature review of the case reports of PE published in the last 24 years and prepared a summary of the characteristics, emphasizing the importance of proper recognition, clinical features, and treatment of this uncommon syndrome.

## 1. Introduction

In 1925, at the 48th meeting of the American Society of Dermatology, Dr Francis Senear and Dr Barney Usher presented patients with an unusual form of pemphigus. They named it pemphigus erythematosus. It remains controversial whether this is pemphigus foliaceus, a subtype of pemphigus foliaceus, a syndrome combining features of lupus erythematosus and pemphigus, or whether Senear–Usher syndrome is a separate disease entity [1]. Senear–Usher syndrome is a very rare autoimmune blistering skin disease with features combining lupus erythematosus and pemphigus foliaceus. The autoantigens in this disease are desmoglein 1, desmoglein 3, and desmosomal adhesion proteins in keratinocytes. The direct immunofluorescence test is positive along the dermal–epidermal junction (DEJ). Antinuclear antibodies (ANA) may be present in 30–80% of patients [2]. The disease most commonly begins in adulthood in patients over 40–50 years of age [3]. Clinically, patients present with skin lesions characteristic of lupus erythematosus and pemphigus foliaceus. On the face, seborrhoeic, discoid lesions develop, mainly in the nasal region, around the nasolabial folds, and on the cheeks, as thick, yellowish, oily scales with a ’butterfly’ distribution. The skin of the body develops flaccid, small vesicles that rupture easily, leaving areas without epidermis. The Nikolsky sign is positive [1]. Involvement of the oral mucosa is very rare. Patients complain of pain, burning, and itching at the site of the skin lesions. Exposure to sunlight may worsen the course of the disease [2]. The overlap of features between lupus erythematosus and pemphigus foliaceus is a diagnostic and therapeutic challenge. Here we present a case report of our patient and review the literature published in the last 24 years describing patients with this entity.

## 2. Case Report

In 2006, a 41-year-old female patient was referred to the Department of Dermatology and Venerology. Numerous, scattered, erythematosus, and scarring lesions with follicular hyperkeratosis were present on the skin of the face and scalp, upper back, and lateral surface of the arms. The skin lesions were not accompanied by itching or pain. Based on the clinical picture and histopathological examination, a disseminated form of discoid lupus erythematosus (DDLE) was diagnosed. Moreover, the patient reported arterial hypertension and gastroesophageal reflux in the medical interview. The family history of dermatological and autoimmune diseases was negative. The patient was treated with oral chloroquine and ointments with topical glucocorticosteroids. Topical steroids were used intermittently from 2016 to 2021. The patient tolerated the treatment well and achieved clinical remission of the disease. For the next 10 years, the patient was under the care of another medical facility.

In 2016, due to the exacerbation of skin lesions in the course of DDLE, diagnostics were extended: ANA titer was determined, and a result of 1:640 was obtained, negative in the specification (Table 1). The serum level of C3 was normal at 1.07 g/L (norm: 0.75–1.4 g/L), and the slightly decreased level of C4 was at 0.08 g/L (norm: 0.09–0.36 g/L). The Coombs test was positive. The patient met the criteria for systemic lupus erythematosus (SLE) according to The Systemic Lupus International Collaborating Clinics (SLICC), satisfying a minimum of four criteria, including at least one of the clinical criteria (chronic cutaneous lesions) and at least one of the immunologic criteria (ANA level above the reference range, reduced C4 complement level, positive Coombs’ test in the absence of haemolytic anaemia) [4]. Due to the predominance of joint problems, methotrexate was added to the treatment at a dose of 12.5 mg per week. The patient reported continued therapy for 6 months. Because of ineffectiveness, the drug was no longer recommended, and the patient was treated with chloroquine (in a dose of 500 mg per day) in accordance with the guidelines of the Polish Dermatological Society, which recommend antimalarial drugs as the first-line general drugs for cutaneous lupus erythematosus [5]. Chloroquine was used from 2016 to September 2021, discontinued due to an increase in liver parameters.

In February 2021, the patient reported an exacerbation of skin lesions, which were associated with COVID-19 infection. The patient was treated as an outpatient at that time, and methylprednisolone at a dose of 32 mg per day was added to the treatment with subsequent dose reduction to 24 mg per day. A slight improvement in skin lesions was observed.

**Figure 1:**
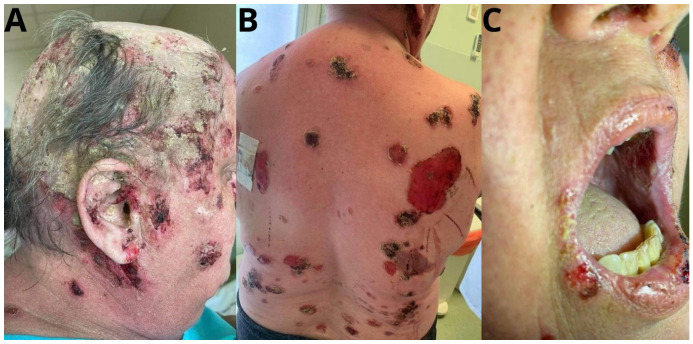
Dermatological examination: (**A**)-Erythematous and erosive eruptions partially covered with scab, yellowish crust disseminated on the scalp and face. (**B**)-Blisters and erosions covered with scabs on the trunk (**C**)-Erosions of the lip mucosa, whitening of the oral mucosa.

However, the patient developed new skin lesions, with a different clinical picture, in July 2021 (Figure 1). Erythematous and erosive eruptions appeared, partially covered with scabs; yellowish crusts disseminated on the trunk and flaccid blisters with serous content. Erosions of the lip mucosa, whitening of the cheek mucosa, as well as scarring in the places of previous skin lesions, were visible. The patient reported pain in the area of oral erosions. ANA test with specification was performed again (ANA 1:320, Scl 70 (+)) (Table 1).

**Figure 2:**
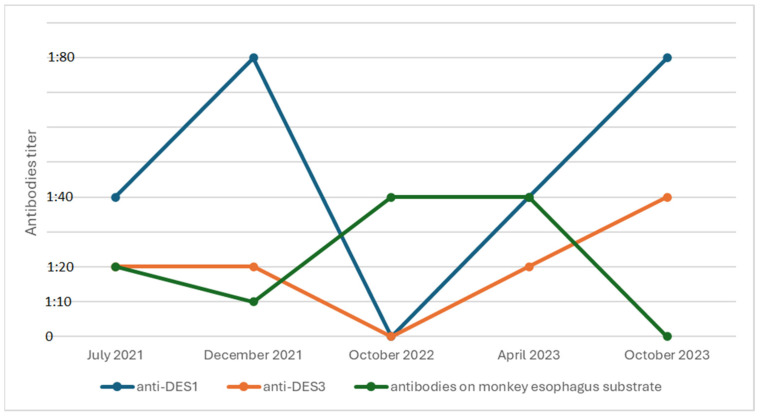
Patient’s antibody follow-up.

Diagnostic tests were extended to tests for autoimmune bullous diseases. Pemphigus/pemphigoid antibodies were determined, obtaining the positive result of anti-desmoglein 1 (anti-DSG1) antibodies 1:40 (consistent with pemphigus erythematosus), anti-desmoglein 3 (anti-DSG3) antibodies 1:20 (BIOCHIP, Euroimmun, Germany), and antibodies on monkey esophagus substrate 1:20 (IIF, Euroimmun, Germany) (Table 1, Figure 2). Direct immunofluorescence (DIF) tests confirmed the presence of deposits of IgG (++) and immunoglobulin A (IgA) (++) in the intercellular spaces of the epidermis. In histopathological examination of skin biopsy, acantholytic epidermis with preserved basal and spinous layers with lymphocyte infiltration in the stroma (Table 1) was described. Based on the clinical picture and the results of additional tests, a diagnosis of pemphigus vulgaris coexisting with SLE was made.

Treatment with prednisone at a dose of 80 mg per day and azathioprine (AZA) at 100 mg per day was started. The patient achieved clinical response. New skin eruptions had not been observed for 5 months. Significant clinical improvement allowed a gradual reduction in the dose of prednisone to 5 mg (the dose of AZA was maintained). Despite the improvement in the clinical picture in December 2021, there was a described increase in anti-DSG-3 titer from 1:40 to 1:80 (Table 1, Figure 2). The patient remained under the care of an outpatient clinic.

**Figure 3:**
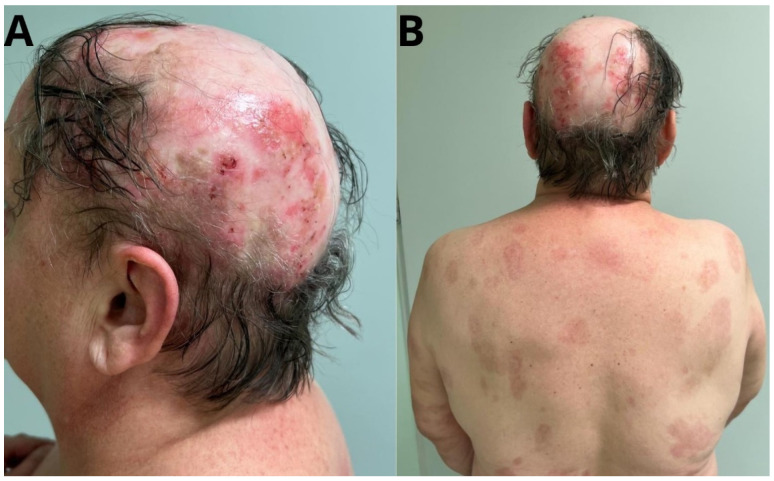
Clinical examination: (**A**)-Scarring lesions with follicular hyperkeratosis, erythematous lesions, and erosions covered with scab. (**B**)-Post-inflammatory discoloration on the skin of the trunk.

In October 2022, a follow-up DES-1 and DES-3 antibody test was performed again, obtaining a negative result (Table 1, Figure 2). The patient continued treatment until April 2023 with satisfactory clinical effect. Then, due to the presence of erosions and new erythematous lesions on the scalp (Figure 3), topical treatment was intensified, whereas AZA (100 mg per day) and prednisone (5 mg per day) were maintained at the previous doses, achieving temporary improvement. The patient reported that she was under strict photoprotection all the time.

**Figure 4:**
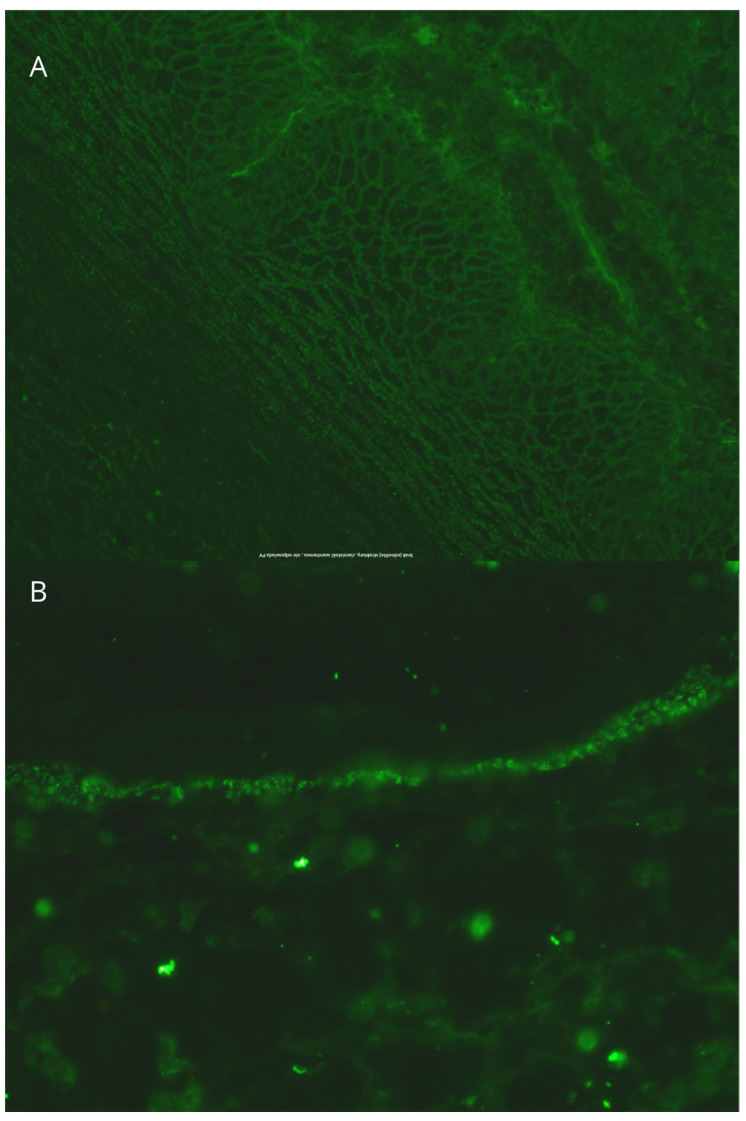
Direct immunofluorescence examination: (**A**)-Direct immunofluorescence is seen with intraepidermal intercellular deposition of granular IgG deposits in the intercellular space of the epidermis. (**B**)-Direct immunofluorescence is seen with Granular deposition of C3 along the dermo–epidermal junction.

Another exacerbation of the disease occurred in October 2023. A follow-up ANA test, pemphigus/pemphigoid antibody tests, and skin samples for DIF and lupus band test (LBT) were taken. DIF showed granular deposits of IgG (+) in the intercellular spaces of the epidermis and granular C3 (+) deposits along the dermo–epidermal junction; the LBT was negative (Table 1, Figure 4). The results of these tests led to the suspicion of PE. Additionally, HCV antibodies were present, whereas HCV RNA was negative. Anti-HBc antibodies were positive, and HBs antigen was negative. The Quantiferon test was negative. A diagnostic imaging was also carried out. A chest X-ray was performed, showing no abnormalities, while an abdominal ultrasound revealed a fatty liver and pancreas, a 9 mm wide cyst in the left kidney, and atheromatic changes in the aorta.

We did not decide to increase the dose of oral glucocorticosteroids due to blood pressure spikes, the development of steroid-induced diabetes, and poor tolerance of steroids in the past (hot flashes). The patient was referred to the Infectious Diseases Department, where the acute phase of hepatitis infection was excluded and entecavir treatment was started. In March 2024, due to insufficient clinical response to the previously used immunosuppressive treatment and chronic side effects of steroid therapy, the patient was qualified for treatment with rituximab (1 g on day one and 1 g on day 15). The patient tolerated the treatment well and remains in clinical remission till the article is published.

Consent was obtained from the study participant to present the case.

## 3. Discussion

Although the described patient did not present typical seborrheic eruptions, skin lesions may appear as circumscribed erythematous erosions, partially covered with scabs [6]. Mucosal involvement is rare [7]. However, in this patient, it was present, as well as erythematous erosions covered with crusts were observed. Typical predilection sites are the trunk, face, scalp, and, less frequently, limbs [1,8]. Two types of lesions may occur on the trunk: small and flaccid blisters and skin lesions covered with scales or scabs with visible seborrheic keratosis, which disappear with hyperpigmentation.

The predominance of PE is observed in women. The progression of the disease is usually slow, which is what we observed in our female patient [9]. Immunological phenomena in patients with PE include features typical of pemphigus foliaceus: the presence of anti-DSG1 antibodies and the erosions on the mucous membranes [3,10]. A typical histopathological picture for PE was observed in this case—the pathologist described the presence of acantholytic epidermis and interface lymphocyte infiltration in the stroma.

The second DIF examination of the described patient showed granular deposits of IgG in the intercellular spaces of the epidermis and granular deposits of the C3 along the basement membrane. Jabłonska and Chorzelski [11] reported that 83% of patients with PE (5 of 6 patients) had IgG deposits along the basement membrane in the skin samples from erythematous lesions from the face. The LBT in our patient was negative. However, the LBT is positive in 70–80% of sun-exposed non-lesional skin specimens obtained from patients with SLE and approximately in 55% of SLE cases when sun-protected, non-lesional skin is analyzed [12]. Our patient used strict photoprotection, which may have influenced the LBT result.

In the literature, many authors reported positive ANA antibodies in patients with PE [11,13]. The described patient had positive ANA antibodies all the time (ranging from 1:80 to 1:640). In the literature, the titer of these antibodies ranged from 1:10 to 1:320, most often with a homogeneous type of fluorescence [14].

Our patients had positive results for both types of antibodies (against DSG1 and DSG3) that were initially detectable. The experiment conducted by P’erez-P’erez et al. [15] also showed the presence of antibodies directed against DSG1 and DSG3 in 8 out of 10 patients with PE. Researchers proposed that this phenomenon may result from epitope spreading, but the role of this phenomenon in the formation of blisters is not yet fully explained.

In the literature, a similar case of Senear–Usher syndrome was described by Chandran et al., who described a patient with significant involvement of the scalp, no characteristic skin lesions in the seborrhoeic area, and no “butterfly” erythema. The patient had antibodies against DSG1 and DSG3, and the DIF revealed IgG and C3 deposition both intercellularly and at the dermo–epidermal junction [16]. Henington et al. [17] described 8 patients with PE, 2 of whom developed acute pemphigus vulgaris. This suggests that Senear–Usher syndrome may appear in the spectrum of pemphigus vulgaris, as it was in our patient.

Using the PubMed database, we performed a descriptive literature review of the English-language case reports and case series applying keywords: ‘Senear Usher syndrome’, ‘Senear Usher’, and ‘pemphigus erythematosus’ published within the last 24 years (2000–2024). After the removal of irrelevant articles based on the abstracts, full-text articles, and papers that did not report a case of PE and those without histopathological examination results and/or DIF, 18 articles were included in this review. Two of the papers were case series without specific clinical information of each patient and were also excluded from the review. Therefore, a total of 16 articles describing 23 patients were included in the literature review, and clinical, histopathological, and immunological information are summarised in Table 2.

The mean age of the patients was 47.39 years with a range of 7 to 80 years. Fourteen of the cases (60.9%) were females and nine (39.1%) were male. The most common locations of skin lesions were the face and trunk, followed by the extremities and scalp. Face involvement was observed in 20 (87%) patients and trunk in 16 (70%) patients. Mucosa involvement was seen only in three patients (13%).

Characteristic skin lesions were usually scaly, crusty, dusky erythematous plaques. Flaccid vesiculobullous, widespread erosions, and scattered erosive erythema were present. In one patient keratotic hyperpigmented papules were described. Histopathologic examination usually revealed acantholysis in the epidermis and inflammatory infiltrate in the dermis consisting of neutrophils and eosinophils. In one patient hyperkeratosis was seen. The intraepithelial blister was described in six patients, whereas the subcorneal blister in six patients.

DIF examination most commonly revealed IgG and C3 deposition (15 of 23 patients, 65.2%) in the intercellular spaces of the epidermis. Fibrin deposits were additionally present in one patient, similarly to IgA deposition, which was also noted in one patient. AlongDEJ, IgG and C3 depositions were also the most commonly reported. However, fibrin, IgM, and IgA were also noted. ANA titter was checked in 15 patients, and in 7 of them (46.7%), it was positive. Autoantibodies against DSG-1 were checked in 14 patients and were elevated in all of them (100%), whereas anti-DSG1 together with anti-DSG3 was present only in 1 patient.

We have also performed a literature review of the case reports describing the coexistence of pemphigus vulgaris and lupus erythematosus, applying the keywords ‘pemphigus vulgaris’ and ‘lupus erythematosus’. Only 4 cases describing the co-occurrence of these two diseases have been published in the last 24 years (2000–2024). The data are summarized in Table 3.

One case report described the coexistence of DLE with pemphigus vulgaris, and the other three described the co-occurrence of SLE with pemphigus vulgaris. The mean age of the patients was 40.5 years with a range of 36 to 46 years. Three of four cases (75%) were females and one (25%) was a man. The involvement of oral mucosa was the main characteristic feature. However, the skin lesions did not exhibit the same pattern of appearance. In the histopathological examination, typical intraepidermal blister and acantholysis were seen, and in the DIF examination, only IgG or IgG with C3 deposition in the intercellular spaces of the epidermis were detected. In one DIF, the deposition along the DEJ was present, which is representative of SLE. ANA was present in 100% of patients, and only 1 of 4 patients was checked for the anti-DSG1 and anti-DSG3 antibodies and had the latter. Some authors tried to differentiate these cases from PE, and the main differentiating features are primarily facial eruptions and bullous lesions on the chest, upper back, and intertriginous areas, and generally minimal clinical signs of lupus erythematosus in cases of PE [18].

## 4. Etiopathogenesis

The prevalence of this disease ranges from 0.5 to 3.2 cases per 100,000 people in the general population. The mode of genetic inheritance remains unclear. It was first described by E. Senear, an American dermatologist, and B. Usher, a Canadian dermatologist. The frequency of the disease is higher among individuals with the HLA A10, DRW6, and A26 alleles [36]. The exact etiopathogenesis of the PE remains unclear. Several external factors can trigger PE in individuals who are genetically predisposed. These factors include drugs, viral infections, and exposure to physical agents such as heat and ultraviolet, as well as surgical and cosmetic procedures [20]. In our literature review, we find two cases of PE associated with drug intake, one with atorvastatin [19] and the second with cefuroxime [20] in a young boy. One case report was triggered by ultraviolet B radiation [28]. Ormsby and Mitchell initially reported the inaugural case of this disease, followed shortly by Senear and Usher, who documented a cohort of 11 patients with PE. These patients exhibited clinical characteristics reminiscent of lupus erythematosus, and biopsy findings revealed acantholysis [37]. The identification of acantholysis and the presence of circulating antibodies in vivo prove that it is an autoimmune disease. Additionally, the detection of immunofluorescence bands in samples from facial and exposed skin, along with the presence of ANA in some cases, indicates overlapping immunological irregularities commonly associated with lupus erythematosus. Therefore, some authors suggest that it is a multiple autoimmune disease [13,38]. Pérez-Pérez et al. examined 10 patients with a diagnosis of PE and the presence of both anti-epithelial and ANA. Because the autoantibodies in the patient’s serum did not show cross-reactivity, the researchers concluded they were produced by independent clones of B cells. Moreover, they disclosed that the desmosome is the immunodominant epitope because the titer of anti-epithelial antibodies was always higher than the antinuclear titer [15].

## 5. Clinical Picture

The Senear–Usher syndrome manifests with two distinctive features. Firstly, facial symptoms include an eruption that can resemble typical lupus erythematosus, with discoid lesions featuring ’carpet tack’ scales in the butterfly area. The second aspect involves body eruptions, which typically start as bullae. These blisters are usually small and flaccid, easily rupturing and leading to the development of two types of lesions: reddish patches and yellowish scale crusts [1]. The disease is usually accompanied by pruritus, stinging, or pain within lesions. While PE has been observed in various age groups, including children and teenagers, it most commonly begins in middle-aged adults, typically appearing in individuals in their forties and fifties [3]. The disease is more often diagnosed in women [9]. As this syndrome is typically described as a coexistence of LE with pemphigus foliaceus, involvement of the oral mucosa is very infrequent [7] in contrast to the co-occurrence of SLE with PV, where the disease usually begins with the presence of erosions on the oral mucosa, and skin lesions appear after several weeks or months [1]. The skin appendages are not affected.

## 6. Diagnosis

The diagnostic criteria of PE contains: the histopathology of PF with DIF findings of intercellular deposits of IgG and/or C3 deposits along the DEJ [9]. Histological findings include acantholysis at the level of the granular layer or below, as well as the formation of blisters in the subcorneal layer of the epidermis. Dermal oedema and perivascular infiltration may also be seen [3,7]. Testing for the presence of antibodies in tissues by DIF is the gold standard in the diagnosis of blistering diseases.

In PE, IgG/IgM and/or complement (C3) deposits are located not only in the intercellular spaces of the epidermis (typical for pemphigus) but also there are granular deposits of IgG or IgM and/or C3 along the dermal–epidermal junction (typical for lupus erythematosus) [7]. The presence of deposits along the basement membrane is important because it supports the diagnosis of PE [27,39]. Lupus band test is a direct immunopathological examination of unaffected skin exposed to UV radiation. In patients with PE, IgG/IgM and/or complement deposits are observed at DEJ [27].

Jabłońska and Chorzelski conducted a study on 54 patients with PE, and 81% of the patients had positive DIF, but only 23% had a positive LBT [13]. Diagnostics also include blood serum tests for the presence of ANA antibodies and antibodies against cell membrane components.

Since PE is considered a variant of pemphigus foliaceus, scientists expect the presence of antibodies against DSG1 in the sera of these patients [40]. DSG1 is a 160 kDa transmembrane protein and is a part of the desmoglein subfamily of the cell adhesion supergene family. Its intracellular domain interacts with plakoglobin, a component of desmosomal plaques, thereby connecting DSG1 to the cytoskeleton for structural support. The extracellular domain of DSG1 contains epitopes relevant to disease and is involved in mediating cell adhesion through heterophilic interactions. This dual functionality emphasizes the critical role of DSG1 in maintaining epidermal integrity and how its dysfunction contributes to the acantholysis [41].

However, patients with PE can have antibodies against DSG3 too. This phenomenon may depend on epitope spreading. It refers to a phenomenon in which the immune system’s recognition of epitopes diversifies over time and can broaden the immune response in autoimmune diseases such as SLE, multiple sclerosis, pemphigus, and bullous pemphigoid [42]. Additionally, the anti-epithelial antibodies, the ANA are also present in patients with PE. In the case of coexistence of SLE and PV, ANA are detected; desmoglein 3 is present in the case of pemphigus vulgaris limited to the mucous membranes or DSG3 and DSG1 in the case of pemphigus of the mucous membranes and skin [16].

## 7. Treatment

Due to the infrequent occurrence of the disease, there are unfortunately no clear therapeutic guidelines and recommendations. The choice of treatment is most often based on the analysis of case reports and disease descriptions. Therefore, the evidence regarding optimal therapy is limited. Because PE is usually considered a mild variant or subtype of pemphigus foliaceus, similar therapies are used as in the latter.

For all types of pemphigus, the generally recommended first-line treatment is systemic corticosteroids (CS). They can be used in monotherapy or in combination. Disease control is usually achieved with low doses (0.5–1 mg/kg/day) of prednisone or its equivalent. Doses generally do not need to exceed 1 mg/kg/day of prednisone, but sometimes as high as 1.5 mg/kg/day are necessary and also acceptable [43,44,45]. Taking high doses of steroids should be continued until new lesions no longer appear. The dose of the medicine can then be gradually lowered to the minimum required to control the disease. In addition to the oral form, glucocorticosteroids such as methylprednisolone can also be used via intravenous administration in the form of pulses. Appropriate monitoring is critical when using steroids. In the case of chronic glucocorticosteroid therapy, the use of proton pump inhibitors or H2 blockers as prophylaxis of peptic ulcer disease, potassium supplementation, and osteoporosis counseling and prevention (vitamin D and calcium intake or even bisphosphonates) should be considered [45,46].

Most often, the group of immunosuppressive drugs other than systemic glucocorticoids used in blistering diseases includes: AZA, mycophenolate mofetil (MMF), cyclophosphamide, and methotrexate (MTX) [24,43,44,45,47,48,49]. The use of these drugs as adjuvants usually is aimed at reducing the dose of systemic steroids and/or shortening the need for their administration, thus reducing the risk of side effects of glucocorticoid therapy. Also, they are recommended when the disease’s course is more severe. As monotherapy, they may be suggested in patients with contraindications to systemic corticosteroid treatment.

The usual doses of AZA are 1–2.5 mg/kg/day. Treatment should start with a lower dose (50 mg/day) and be gradually increased with simultaneous control of blood counts and thiopurine methyltransferase (TPMT) activity to avoid adverse effects such as bone marrow toxicity (when TPMT serum level is low or intermediate) or, on the contrary, to avoid lack of therapeutic effectiveness (when TPMT serum level is very high). The disadvantage of AZA is its delayed action because it has an immunosuppressive effect only 6–8 weeks after starting therapy [45,50]. Mycophenolate mofetil shows similar tolerance, safety, and efficacy to AZA in patients with pemphigus foliaceus [51].

Cases of effective use of hydroxychloroquine [52] or dapsone [45,53,54] in addition to immunosuppressive drugs have also been reported. Using dapsone allowed for a reduction in the daily dose of CS in patients with stable disease, and the improvement was visible after just a few weeks of therapy. However, one should remember the possible side effects, such as methemoglobinemia. Contraindications to the use of dapsone are confirmed drug hypersensitivity, anemia, and deficiency of glucose-6-phosphate dehydrogenase (G-6-PD) [55]. Campolmi et al. reported three patients with recurrence of PE despite maintenance dose steroid therapy who achieved remission when cyclosporine A (5 mg/kg/day) was added to prednisone (1 mg/kg/day) [56].

According to the literature, other effective but less frequently used drugs or procedures in the therapy of PE are: tetracycline plus niacinamide, plasmapheresis, or intravenous infusion of immunoglobulin [45,48,57,58]. Some biological drugs also appear promising in the treatment of PE. These data are based on case series studies. The benefits of these drugs are usually seen in steroid-dependent patients, or in patients who do not respond to oral corticosteroid-sparing drugs, or those who do not respond.

Rituximab is a chimeric anti-CD20 monoclonal antibody targeting pre-B cells and mature B cells. It is approved by the Food and Drug Administration (FDA) for the treatment of pemphigus vulgaris, and it may be used as a single agent or in combination with other immunosuppressants to taper their doses [45,48,59]. Moreover, several clinical trials, such as the EXPLORER study and the RITUXILUP study, have shown that rituximab in SLE can help reduce disease activity in selected groups, improve renal outcomes, and reduce the need for steroids [60,61,62].Therefore, it seems to be a good choice in patients with coexistence of SLE with PV or PF. De Vries et al. described a case of a patient with Senear–Usher syndrome who did not initially respond to treatment with systemic prednisone and MMF but achieved remission of the disease after the use of rituximab as an adjuvant therapy [29]. Due to its strong immunosuppressive properties, the administration of rituximab should be combined with a reduction in the previously used doses of glucocorticosteroids and other immunosuppressive drugs. Severe side effects associated with the use of rituximab, such as progressive multifocal leukoencephalopathy, serious infusion-related reactions (e.g., anaphylaxis), hypogammaglobulinemia, and life-threatening infections due to long-lasting B-cell depletion, which impairs the ability to respond to certain pathogens, are rare, although invasive fungal infections and reactivation of latent infections (e.g., hepatitis B, tuberculosis) have been reported [63,64,65,66]. Therefore, it is recommended to perform infection-screening before starting treatment.

The use of another monoclonal humanized anti-CD20 antibody—veltuzumab has also shown promising results [2]. Recently, two case reports revealed successful usage of dupilumab (a human monoclonal antibody directed against the IL-4 alpha receptor that inhibits the signaling of IL-4 and IL-13) in the treatment of PE. Both patients were managed with glucocorticosteroid in combination with dupilumab, resulting in symptoms diminished and providing relief from itching [21]. Infliximab, adalimumab, and etanercept—Tumor Necrosis Factor alpha (TNF-α) inhibitors, have also been tested, but data are limited [48].

Topical treatment is usually used as a complementary therapy to systemic drugs, or it may be sufficient as the only form of treatment in very mild cases or in localized forms of disease [45,67,68].

In PE, topical steroids are most commonly chosen. The strength, dose, and form of the drug depend on various factors such as the patient’s age, location of skin lesions, characteristics of lesions, and potential risk of steroid adverse 353 effects [69]. With long-term local treatment, glucocorticosteroids may be replaced with topical calcineurin inhibitors; tacrolimus or pimecrolimus are used less frequently from the beginning of therapy [67,69,70]. Other drugs supporting the treatment of pemphigus may be local analgesics and antiseptics. We should not forget to recommend photoprotection, including the use of UV protective clothing and daily sunscreen, because sunlight may exacerbate disease activity [25].

Treatment should be carried out by a dermatologist experienced in the treatment of pemphigus in close cooperation with a clinical center, where laboratory diagnostics and treatment control will be possible because the disease is chronic and recurrent. Monitoring should include the patient’s clinical condition, immunological parameters of the disease, and the occurrence of potential side effects of the drugs used, which will ultimately enable the assessment of the effectiveness and safety of the therapy.

## 8. Conclusions

This case can be described as a co-occurrence of multiple autoimmune processes. Considering the clinical picture, results of immunological tests, and histopathological examination, we can treat this patient as an atypical case of Senear–Usher syndrome, in the spectrum of pemphigus vulgaris with involvement of mucous membranes. In our opinion, the patient clinically more closely resembles pemphigus erythematosus (erythematous and erosive eruptions, covered with scab, yellowish crusts, and flaccid blisters in typical localization) and is consistent with other characteristic features of patients with PE shown in Table 2. Moreover, systemic symptoms of SLE were not very pronounced in this patient. The diagnostic (DIF, indirect immunofluorescence, histopathology, LBT, ANA) and therapeutic (topical CS, systemic CS, AZA, MMF, MTX, cyclophosphamide, hydroxychlorochine, dapsone, and biological drugs) strategies presented in this article are intended to reduce the underestimation of this syndrome incidence and to facilitate therapeutic decisions for clinicians who encounter this rare disease entity. This review emphasizes the importance of an individualized diagnostic approach in complex autoimmune skin diseases and indicates the need to develop targeted therapies in the future, which will not only be more effective but also have a lower risk of side effects. The conclusions of this case report may also foster academic discussion and inspire further exploration of the nuanced presentations of chronic inflammatory skin diseases.

## Figures and Tables

**Table 1:** Results of diagnostic tests. ND—no date.

Date	2016	July 2021	December 2021	October 2022	April 2023	October 2023
ANA titer and type of fluorescence	1:640 speckled-granular and mitochondrial	1:320 nuclear, 1:320 granular	ND	ND	ND	1:320 nuclear, 1:160 granular, 1:80 cytoplasmic
ANA specificity	negative	Scl 70 (+)	ND	ND	ND	ND
anti-DSG1 antibodies titer	ND	1:40	1:80	negative	1:40	1:80
anti-DSG3 antibodies titer	ND	1:20	1:20	negative	1:20	1:40
Antibodies on monkey esophagus substrate	ND	1:20	1:10	1:40	1:40	negative
DIF	ND	deposits of IgG (++) and IgA (++) in the intercellular spaces of the epidermis	ND	ND	ND	granular deposits of IgG (+) in the intercellular spaces of the epidermis, granular C3 (+) deposits along the dermo–epidermal junction
LBT	ND	ND	ND	ND	ND	negative
Histopathology	ND	skin section covered with acantholytic epidermis with preserved basal and spinous layers (bottom of the blister) with lymphocyte infiltration in the stroma—the image may correspond to the diagnosis of pemphigus	ND	ND	ND	ND

**Table 2:** Clinical and serological features of patients with PE between 2000 and 2024. f, female; m, male; DIF-ICS, direct-immunofluorescence-intercellular space staining; DIF-DEJ, direct-immunofluorescence-dermo–epidermal junction; DLE, discoid lupus erythematosus; IgM, immunoglobulin M; NA, not available; “+” positive; “−” negative.

Study Year	Demographic	Location of Lesions	Appearance of Skin Lesions	Histopathological Examination	DIF-ICS	DIF-DEJ	ANA	DSG-1	DSG-3
Hobbs, 2021 [9]	37, f	scalp and face	thick, scaly, crusty erythematous plaques	epidermis: acantholysis dermis: perivascular and interstitial infiltrate	IgG	granular IgG and C3, fibrin (focal) deposition	−	NA	NA
Hobbs, 2021 [9]	44, f	face and oral mucosa	heavy scale, underlying erythema without blisters or erosions	epidermis: acantholysis dermis: inflammatory infiltrate	−	IgG, C3, fibrin around dermal vessels	−	−	NA
Hobbs, 2021 [9]	68, m	trunk and scalp	classic raw erosive changes with some crusting	epidermis: acantholysis, dermis: perivascular infiltrate	IgG, C3, fibrin	C3	NA	NA	NA
Hobbs, 2021 [9]	53, f	face	scaly, dusky plaques with erythematous raised borders	epidermis: keratinocyte necrosis and vacuolar alteration of the basal cell layer along with apoptotic bodies, dermis: perivascular infiltrate	IgG, C3	granular C3, focal IgM, granular IgA	+	+	NA
Hobbs, 2021 [9]	17, m	face, extremities, trunk, and oral mucosa	crusted, vegetative plaques, intact tense small bullae and vesicles	epidermis: acantholysis	IgG, C3	granular C3	NA	NA	NA
Garcia-Souto, 2020 [18]	53, f	face	crusted erosions over the face	intraepidermal blister with acantholysis of the granular layer	IgG, C3	−	NA	NA	NA
Lo Schiavo, 2014 [19]	70, m	face and trunk	erythematous scaly plaques which involved the cheeks in a butterfly distribution symmetrically and crusted lesions localized on the upper part of the chest	intraepithelial superficial blister	IgG, C3	IgG, C3	+	+	NA
Neha Chandan, 2018 [16]	24, f	trunk, extremities, and face	erythematous, eroded, boggy scalp, crusting, and scale adherent to the residual hair, with yellow to brown debris	suprabasal and intraepidermal acantholysis with no interface or basal vacuolar changes	IgG	IgG, C3	−	+	+
Baroni, 2009 [20]	14, m	scalp	flaccid vesiculobullae, erosions, and crusted lesions over the upper part of the chest and face, and diffuse scaly plaques on the scalp	intraepidermal superficial blistering containing a few acantholytic cells	IgG, C3	−	NA	+	−
Chen, 2023 [21]	39, m	face, trunk, and extremities	extensive blistering and ulceration on the face, trunk, abdomen, and extremities	intraepithelial cleavage with detached keratinocytes primarily localized just under the stratum corneum	IgG, C3	−	NA	NA	NA
Chen, 2023 [21]	59, f	face, trunk, and extremities	widespread erosions affecting the face, trunk, abdomen, and limbs	subcorneal split directly below the stratum corneum	IgG, C3	−	NA	NA	NA
Bilgic, 2018 [22]	54, m	face, scalp	well-demarcated, erythematous -squamous plaques, some with atrophic center or with cicatricial alopecia, on the scalp, nose, malar area and lips, bullae, and erosions with scale crusts on the trunk	compatible with DLE and PF	IgG	−	−	+	−
Sawamura, 2016 [23]	65, f	face, trunk, and extremities	scattered erosive erythema with scaling and crusting throughout the body including the face	intraepidermal blisters containing neutrophils and acantholytic keratinocytes	IgG	IgG	NA	+	−
Gupta, 2004 [24]	7, m	extremities, face, scalp	generalized scaling and redness associated with edema of the upper and lower extremities, flaccid blisters on the scalp, face, and both lower extremities	compatible with pemphigus erythematosus	IgG, C3	IgG, C3	−	+	−
Pritchett, 2015 [25]	40, f	face and trunk	pruritic, pink scaling plaques on her face and neck, lesions subsequently involved chest, abdomen, and back, and flared with sun exposure	epidermal acanthosis, spongiosis, and acantholytic keratinocytes lymphoplasmacellular infiltrate was seen in the dermis, with scattered neutrophils and eosinophils, foci of eosinophilic spongiosis in the epidermis	IgG, C3	IgG, C3	+	NA	NA
Diab, 2008 [26]	18, f	face, trunk, extremities	keratotic hyperpigmented papules and plaques on the face, upper trunk, and proximal extremities	acantholysis within the granular cell layer with concomitant interface dermatitis	IgG, C3	IgM	NA	+	NA
Dyah, 2012 [27]	80, f	face, trunk, extremities	generalized progressive erythematous skin lesions with pustules and flaccid blisters	subcorneal blisters	IgG, C3	−	−	+	−
Dyah, 2012 [27]	76, f	face, scalp, trunk, extremities	itching plaques all over body and scalp except her legs	ulcerative and erosive inflammation and secondary impetigo with beginning reepithelialization	IgG, C3	IgG, C3, IgM	−	+	−
Dyah, 2012 [27]	68, m	scalp, extremities, trunk	red scaly skin lesions starting on the face, chest, and back, blisters on the whole body, including the scalp and extremities	remainder of a blister in the corneal layer and subepidermal neutrophilic infiltrates surrounding the blood vessels	IgG, IgA, C3	IgG, IgA, and C3	−	+	−
Makino, 2014 [28]	62, f	face, trunk, extremities	pruritic and slightly erythematous lesions with erosions on the nose, chest, and extremities	detachment of the stratum corneum, infiltration of lymphocytes	IgG	IgM, C3	+	+	−
de Vries, 2023 [29]	51, m	face, scalp, and trunk	pruritic scaly plaques on the face, scalp, and trunk	acantholysis and granular layer separation	NA	NA	+	+	−
Shankar, 2004 [30]	59, f	face, trunk, scalp, extremities	pruritic rash on the upper chest and upper back, face, scalp and thighs	acantholysis of the superficial epidermis with the formation of a subcorneal bulla and a very mild patchy interface dermatitis	IgG, C3	IgM, C3	+	NA	NA
Chavan, 2013 [31]	32, f	face, scalp, trunk, extremities, oral mucosa	erythematous papules over cheeks, ears, scalp, upper back, “V” of the chest and extensors of forearms and dorsal of hands, crusted scaly, plaques with surrounding erythema, erosions over upper gingiva	hyperkeratosis, subcorneal acantholysis, basal cell vacuolation, dermo–epidermal separation and superficial perivascular lymphocytic infiltrate	IgG	IgM, IgG	+	NA	NA

**Table 3:** Characteristics, clinical and serological features of patients with pemphigus vulgaris and SLE between 2000 and 2024.

Study, Year	Demographic	Location of Lesions	Appearance of Skin Lesions	Histopathological Examination	DIF-ICS	DIF-DEJ	ANA	DSG-1	DSG-3
Hidalgo, 2001 [32]	46, m	chest, extremities	pruritic eruption of symmetric lesions of 0.5–1 cm in diameter, consisting of vesicles on the chest, back, shoulders and legs	intraepidermal detachment forming blisters and fissures in a suprabasal portion of the epidermis, with acantholysis, next to spongiform vesicles filled with polymorphs and several eosinophils	IgG, C3	−	+	NA	NA
Thongprasom, 2013 [33]	36, f	oral mucosa, face	desquamative gingivitis, erythematous facial rash with acne-like papules	acantholysis of the epithelial cells, which exhibited intraepithelial separation, particularly in the lower spinous cell layer and perivascular infiltration of lymphocytes in the lamina propria	IgG	−	+	NA	NA
Nanda, 2004 [34]	45, f	oral mucosa	ulcers on the buccal mucosa and soft palate with inflammation of the gums and soft pharynx	intraepithelial blister with acantholytic cells	IgG, C3	−	+	−	+
Calebotta, 2004 [35]	35, f	extremities, face, and oral mucosa	generalized multiple erosions with zero-hematic crusts on the trunk and both legs, flaccid bullae with serous content located on the thigh, multiple erosions on oral mucosa	separation in a plane above the basal layer of the epidermis	IgG	IgM, C3	+	NA	NA

## Data Availability

Data sharing is not applicable.
